# Emotion Detection Using Deep Normalized Attention-Based Neural Network and Modified-Random Forest

**DOI:** 10.3390/s23010225

**Published:** 2022-12-26

**Authors:** Shtwai Alsubai

**Affiliations:** College of Computer Engineering and Sciences, Prince Sattam bin Abdulaziz University, Al-Kharj 11942, Saudi Arabia; sa.alsubai@psau.edu.sa

**Keywords:** emotion detection, electroencephalogram, deep normalized attention-based residual convolutional neural network, modified-random forest

## Abstract

In the contemporary world, emotion detection of humans is procuring huge scope in extensive dimensions such as bio-metric security, HCI (human–computer interaction), etc. Such emotions could be detected from various means, such as information integration from facial expressions, gestures, speech, etc. Though such physical depictions contribute to emotion detection, EEG (electroencephalogram) signals have gained significant focus in emotion detection due to their sensitivity to alterations in emotional states. Hence, such signals could explore significant emotional state features. However, manual detection from EEG signals is a time-consuming process. With the evolution of artificial intelligence, researchers have attempted to use different data mining algorithms for emotion detection from EEG signals. Nevertheless, they have shown ineffective accuracy. To resolve this, the present study proposes a DNA-RCNN (Deep Normalized Attention-based Residual Convolutional Neural Network) to extract the appropriate features based on the discriminative representation of features. The proposed NN also explores alluring features with the proposed attention modules leading to consistent performance. Finally, classification is performed by the proposed M-RF (modified-random forest) with an empirical loss function. In this process, the learning weights on the data subset alleviate loss amongst the predicted value and ground truth, which assists in precise classification. Performance and comparative analysis are considered to explore the better performance of the proposed system in detecting emotions from EEG signals that confirms its effectiveness.

## 1. Introduction

Emotions greatly impact an individual’s social skills and view of the world. In recent years, several intelligent systems have begun to utilize emotion recognition frameworks to enhance their interactivity with humans. This process is significant as systems could acclimatize their behavioral patterns and responses under human emotions and make interactions natural. Such emotions could also be identified by additional factors encompassing speech, physiological signals, and expressions. Among several factors, EEG (electroencephalogram) signals have proven to possess strong associations with emotional states and have maximum accuracy for classifying emotions. It also reacts more quickly to emotional alterations. Hence, evaluating EEG signals have turned efficient and reliable in accomplishing emotion recognition [1]. However, these are internal brain signals that individuals cannot control. Due to this factor, challenges have grown for certain individuals such as psychologists and CS (Computer Science) researchers in accurately determining feelings from EEG signals. In addition, the noise in the EEG signal also intrudes on identifying an individual’s emotions from EEG signals. In low-frequency bands, data processing becomes complex, which in turn affects the process of noise removal [2]. Presently, advanced ML (machine learning) and DL (deep learning) algorithms have been accessible that could extract and control activities or features for emotion detection from different data types like images. Using suitable ML and DL algorithms based on needs and purpose could assist in attaining reliable outcomes for detecting emotions [3].

Extracting suitable features from the EEG signals is one of the main tasks in correct emotion detection. Considering this, the research [4] has used local-ternary patterns for extracting relevant features from the EEG signals. High and low features have been extracted through the recommended dimension-ternary patterns. Classification has been performed by certain ML methodologies, namely SVM (Support Vector Machine), BayesNet (Bayes Networks), ANN (Artificial Neural Network), and RF (Random Forest). Evaluation outcomes have exposed the better performance of RF. In addition, a hybrid feature extraction approach has been considered [5]. First, asymmetry in varied brain areas has been captured in a two-dimensional vector called AsMap from differential features of the EEG signals. Then, such maps were utilized for extracting features automatically through a CNN (Convolutional Neural Network) model. The suggested feature extraction methodology has been analyzed by comparing it with DE (Differential Entropy) and subsequent feature extraction approaches such as relative asymmetry, differential causality, and differential asymmetry. Experiments have been undertaken, and outcomes have explored the satisfactory performance of the endorsed system. It has also emphasized the significance of feature extraction through a hybrid approach, as the classifier’s detection rate is directly dependent on feature quality. Outcomes have exposed that a hybrid approach of automated and manual feature extraction has shown better performance than the conventional system for recognizing emotions from EEG. 

However, different approaches have been considered for recognizing emotions. Accordingly, LSTM has been used to perform epileptic EEG classification through an optimal wavelet algorithm that relied on *p*-value and correlation value. Additionally, DWT (Discrete Wavelet Transform) has been applied for noise removal to extract 20 eigenvalues (as features). Then, optimal features have been selected through *p*-value and correlation analysis. The suggested system has exposed significant minimization in trainable parameters required for procuring a better detection rate.

Furthermore, PCA (Principal Component Analysis) and CCP (Correlation Coefficient and *p*-value feature) have been employed to minimize features for the complexity reduction of LSTM [6]. Additionally, a supervised ML model has been recommended for recognizing humans’ inner emotions in a two-dimensional framework. EEG signals have been considered to detect emotions. DWT has been employed on the pre-processed signals for extracting desired five frequency bands. A few features such as energy, time domain, DE, and power have been extracted. In addition, channel-wise SVM has been developed. A channel combiner has also been performed to determine appropriate emotion states. The maximum classification performance rate among the four classes is 86% [7]. Though conventional studies have shown better outcomes, accuracy has to be enhanced further for optimal emotion detection. To accomplish this, the present research intends to propose suitable data mining algorithms for attaining high accuracy in emotion detection from the EEG signals.

The main contributions of this study are:

1. To perform feature extraction through the proposed DNA-RCNN (Deep Normalized Attention-based Residual Convolutional Neural Network) for extracting relevant features based on the discriminative representation of features.
2. To internally validate the classification performance of the proposed M-RF (Modified-Random Forest) with NB (Naïve Bayes), DT (Decision Tree), and K-NN (K-Nearest Neighbor) for exploring the effective performing model in emotion classification.
3. To validate the proposed system by comparative evaluation with the conventional system for confirming the proposed system’s effective performance in detecting emotions.

The paper is organized as follows: Section 2 discusses the conventional works and problems; Section 3 describes the proposed system with the relevant flow, algorithm, and description; and Section 4 summarizes the results procured after the execution of the proposed system that encompasses dataset description, performance metrics, outcomes of performance and comparative analysis. Finally, the overall research is summarized in Section 5 with future recommendations.

## 2. Review of Existing Work

Conventional researchers have considered varied data mining algorithms for emotion detection. The complementary approaches are discussed in this section.

Study [8] used a data mining method for recognizing emotions from EEG signals. In addition, a sparse learning-based feature selection technique has been regarded. An entropy-weighted clustering has been integrated with features attained after the sparse learning process. The suggested approach has classified emotions as negative and positive, with 68.35% as the prediction rate, which exposes its better performance. Recall and accuracy have been considered performance metrics to assess varied classification methodologies while recognizing emotions. TP (True Positive) and FP (False Positive) have also been regarded. Taking into account the vital statistical features integrated with the bi-partition labeling method, emotions could be distinguished in a better manner. Accordingly, the ability of SVM has been assessed for detecting emotions while integrating with data mining methods. Outcomes have exposed that the considered SVM (Support Vector Machine) algorithm has performed better in comparison to NB (Naïve Bayes) [9]. To minimize the signal’s dimensionality, the research [10] has contributed to the successful execution of spatial PCA for selecting suitable features under t-statistical interpretations. After extracting suitable features, four classifiers—namely SVM, LDA (Linear Discriminant Analysis), K-NN (K-Nearest Neighbor), and ANN (Artificial Neural Network)—have been employed for classifying emotional states. Recommended system has exposed 77.1% and 84.3% with SVM and ANN [11]. To enhance the classification rate, dimensionality reduction [12] and signal decomposition have been integrated while minimizing the time complexity. 

In addition, a method for feature extraction has been suggested that has fused LDA and differential entropy. Five classification models have been considered for classification, namely LR (Logistic Regression), SVM, K-NN, MLP (Multi-Layer Perceptron), and RF (Random Forest). An average accuracy of 68% has been attained. Execution time has exposed that the suggested system has minimum time complexity. Furthermore, signal processing approaches and optimized SVM with genetic algorithm have been employed to develop the smart methodology for enhancing emotion detection. Attained outcomes have explored that, the recommended system has revealed 93.86% accuracy [13,14]. A multichannel feature fusion approach has been considered for recognizing varied human emotions. A wavelet transform has been leveraged for attaining high-dimensional EEG integrating features to recognize emotions. Empirical outcomes have shown that, in comparison to single signal emotion recognition, the recommended multichannel approach has accomplished better performance about accuracy [15].

Furthermore, a median filter has been utilized to enhance the classification performance to eliminate false detections that have exposed 86.56% accuracy [16]. Moreover, under the development of feature maps, a model has been endorsed for detecting emotions. Feature maps have been relying on HOLO-FM (Holographic) and TOPO-M (Topographic) indications of EEG [17]. DL has been considered a method for feature extraction. Subsequently, extracted features have been integrated for classification to recognize different emotions. As a result, better outcomes have been attained. In addition, conventional ML and DL models have been regarded to classify emotions based on EEG signals. It has been concluded that, under the frequency and time integration of EEG features, the suggested CNN (Convolutional Neural Network) model that has been identical to that considered for classification in computer vision can automatically learn discriminant stimulus associated with EEG dynamics [18]. To improve the accuracy rate, the study [19] has employed TQWT (Tunable Q-Wavelet Transform) integrated with RFE (Rotation Forest Ensemble) in classifying emotions which has attained 93.1% accuracy.

Similarly, an automated framework has been considered to identify emotions. The suggested system has relied on three varied methodologies: VMD (Variational Mode Decomposition) and IMF/EMD (Intrinsic Mode Function/Empirical Mode Decomposition) for noise elimination from signals and to attain better details from EEG data. In addition, four major classifiers have been used for classification: K-NN, DT (Decision Tree), CNN, and NB (Naïve Bayes). 

Nevertheless, CNN has performed better than conventional techniques in categorizing EEG signals [20]. Besides, for concurrent learning of features and recognizing emotions, the study [21] has used a CNN model [22] that relies on SEED (SJTU Emotion EEG Dataset) with Adam optimizer and ResNet-50. Accuracy has been exposed to 94.13%. Furthermore, a DL framework has been explored for accomplishing subject-independent recognition of emotions that encompasses two main phases. Initially, an unsupervised LSTM with channel attention AE (Autoencoder) has been endorsed. Subsequently, CNN has been applied with the attention model to perform emotion recognition independent of the subject. As a result, a maximum of 76.7% accuracy has been attained [23]. Likewise, for considering long-term emotional interactions, the research [24] has endorsed LSTM for considering alterations in emotions and employing an attention mechanism for assigning weights to emotional states at particular moments. Outcomes have exposed that the accuracy rate has been 87.9% and 90.1% through two-stage classification.

The study [25] also intended to process EEG signals based on DL networks without transforming to any domains for determining the emotional state. To accomplish this, EEG recordings have been transformed into patterns of dimension (28*28) in the initial phase. Subsequently, the matrix of the pattern segment developed for three varied emotional states has been integrated into the process of CNN architecture. Finally, segments of EEG have been trained on CNN and tested five-fold. Procured outcomes have been compared with relevant research showing 84.50% while considering ANN and 83.77% as success rates while using K-NN. Thus, it has been clear that the considered DL-based algorithms have shown better performance. Taking this better-performing ability of DL into account, a DL-based approach has been used [26].

On the contrary, the conventional emotion recognition model, DE (Differential Entropy), has been extracted from the individual partitioned segment for generating a feature cube. Multiple GCNNs have been employed for extracting graph-domain features from individual feature cubes. Additionally, LSTM cells have been employed for memorizing the relationship alterations amongst two channels of EEG for extracting temporal features. Accuracy has been exposed to be 90.60% and 90.45% for arousal and valence in experiments dependent on the subject. 

Similarly, a data-adaptable CNN model has been designed to enhance accuracy while classifying emotions. An efficient spectrogram-feature extraction model has been executed and a multi-model classifier has been suggested which considers dual features that have accomplished 73.4% accuracy [27]. Likewise, the research [28] has presented multiple-column CNN for recognizing emotions from EEG. Comparison has explored the better performance of the recommended model. In addition, an SAE (Stacked Autoencoder) model has been utilized for constructing and resolving linear EEG models and emotion timing that relies on LSTM-RNN (LSTM-Recurrent Neural Network). Finally, the framework has been executed on the DEAP dataset to recognize emotions wherein the mean prediction rate of emotions has accomplished at 74.38% (in arousal) and 81.10% (in valence) [29,30].

Similarly, the research [31] has considered an emotion recognition system based on EEG. Comprehensive feature sets have been extracted from the EEG signal. Quantitative analysis has also been undertaken by comparing feature extraction methodologies using three varied ML classifiers: DT, K-NN, and SVM. It has been found that the suggested system has accomplished 77.62% accuracy for valence classification, 78.96% for arousal classification, and 77.60% for dominance classification. Moreover, the application of variations of VGG-16 and ResNet-18 on classifying plain-EEG signals has been regarded for recognizing emotions. Performance has been compared with variants of VGG. Accuracy has been explored to be 93.42% [32].

Significant issues determined through the evaluation of the above conventional works are exposed in this section.

(i) Conventional works have attained a better accuracy rate in recognizing emotions from EEG signs using different data mining approaches. Accordingly, an entropy-weighted clustering approach has been integrated with sparse learning has exposed 68.35% [8], and ANN has shown 84.3%. SVM has achieved 77.1% accuracy [10], while Rotation Forest-SVM has attained 93.1% [19], LSTM with attention Autoencoder has achieved 76.7% [23], optimized SVM reached 93.86% [13], K-NN reached 83.77%, and ANN reached 84.50% [25], and CNN has attained 94.13% [21]. Though better performance has been attained, the accuracy rate has to be enhanced for effective emotion detection [11].
(ii) Varied DL-based models could be utilized for dynamic modeling and feature extraction to enhance performance [12].
(iii) Further research must examine alterations to the emotion analysis model by integrating multiple NNs. Another strategy is enhancing the concatenation of convolution layers. There might be a possibility for constructing convolutional layers for individual data features to enhance classification performance through several convolution layers [27].

## 3. Proposed Methodology

**Figure 1:**
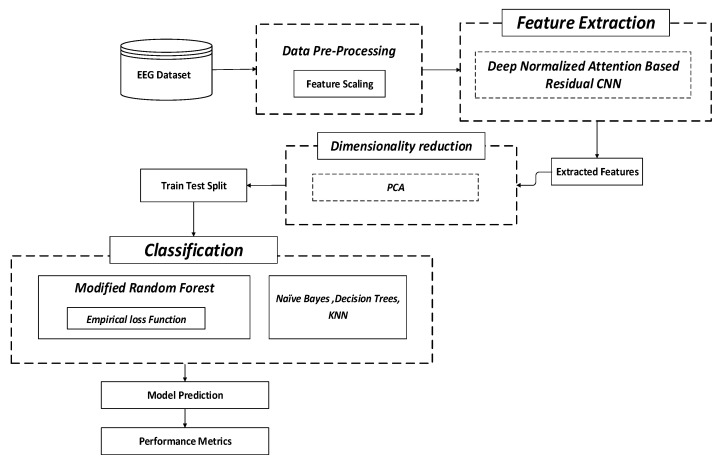
Overall view of the proposed system.

The research aims to achieve emotion detection through suitable data mining algorithms. Though traditional works have endeavoured to perform this, they could have been more effective concerning accuracy rate. To mitigate this pitfall, the study proposes different algorithms for pre-processing the data, feature extraction, dimensionality reduction, and classification, as shown in Figure 1. In this study, DNA-RCNN is proposed for feature extraction, PCA (Principal Component Analysis) is considered for dimensionality reduction and M-RF is proposed for classification with other classifiers such as NB, DT, and K-NN.

At first, the EEG dataset is taken, and pre-processing is performed. In this context, feature scaling is performed in the pre-processing stage. This approach is used for normalizing the independent variable range or data features. Following this, feature extraction is undertaken using DNA-RCNN. This process assists in minimizing redundant data. Such data reduction supports constructing the model with minimum efforts of the machine that also enhances the learning speed as well as the generalization stages in the ML process. Following this, dimensionality reduction is undertaken using PCA (Principal Component Analysis), which determines the minimum or eliminates the least significant variables from the model. This process will alleviate the complexity of the model and remove data noise. By this, dimensionality reduction supports mitigating overfitting. Finally, classification is accomplished by M-RF based on the empirical loss function. Furthermore, through the use of the proposed classifier with an empirical loss function on RF (Random Forest) is capable of affording reliable and accurate outcomes in comparison with conventional algorithms due to the optimized loss-variations undertaken with the proposed system. In this process, other ML classifiers (NB, K-NN, and DT) are also considered for classification to assess the performance of proposed M-RF internally and considered ML classifiers. The overall performance of the model is assessed through performance metrics.

### 3.1. Feature Extraction Using DNA-RCNN (Deep Normalized Attention-Based Residual Convolutional Neural Network)

Generally, CNN possesses the feature of considering significant and clear features at the sight line, so it is extensively utilized in feature extraction. Weight sharing and local perception of CNN could greatly minimize the parameters, thereby enhancing the efficacy of the learning models. Moreover, CNN comprises three main parts: convolution, pooling, and FC (Fully Connected) layer. The individual convolution layer encompasses the plurality of the convolution kernel. When the convolution function of the convolution layer succeeds, data features are extracted. This study additionally proposes Attention Residual CNN for training deep attention networks that could be easily scaled to hundred layers. In addition to several discriminative feature indications created by the attention mechanism, the proposed model also explores appealing features such as enhancing the attention modules resulting in consistent enhancement. Stacking the attention modules might have a direct negative impact on performance. Thus, this study proposes DNA-RCNN with CNN for optimizing deep network with several layers wherein the output of attention module AM is modified as per Equation (1),

(1) $${\mathrm{AM}}_{i,c}\left(a\right)=\left(1+N_{i,c}\left(a\right)\right)*{\mathrm{OF}}_{i,c}\left(a\right)$$

where N(a) is from (0, 1), N (a) = 0, AM (a) indicates original features—F(a). This method is termed attention-residual learning. The proposed attention-residual learning varies from residual learning. In the actual ResNet, the residual leaning is given by Equation (2),

(2) $${\mathrm{AM}}_{i,c}\left(a\right)=a+{\;\mathrm{OF}}_{i,c}\left(a\right)$$

where $F_{i,c}\left(a\right)$ indicates residual function. In the formulation of the present study, ${\mathrm{OF}}_{i,\;c}\left(a\right)$ represent features produced by Deep CNN.

### 3.2. Dimensionality Reduction through PCA (Principal Component Analysis)

PCA is typically a renowned statistical strategy and an orthogonal-linear transformation that is considered for minimizing dataset dimensionality. This algorithm performs dataset transformation into a new system. Maximum data point variance in a new system through any data object projection lies on the initial coordinate, termed an ‘initial principal component’. Subsequent variance is termed the second-principal component, and this process persists. By this process, PCA transforms the data matrix (Y) of size (a∗b) into a minimized matrix (Z) of size (a∗c) for dimensionality reduction, wherein (c<b).

The main notion of PCA is that, with less information based on linear transformation, actual multi-dimensional data variants can be altered with few sovereign principal components. The original sample nature could be explored by the principal components having significance. For instance, alteration in the actual data is reflected by the initial principal component, and the remaining principal components could replicate other features of the sample. Major PCA phases are,

1. 1. Primitive data standardization for eliminating adverse effects instigated by varied dimensions. Actual data are given by Equation (3),

(3) $$A={\left(a_{\mathrm{ij}}\right)}_{n*p}=\left(A_{1},A_{2},A_{3},\ldots \ldots ..A_{P}\right)$$

The formula is standardized as per the Equation (4),

(4) $$G_{\mathrm{ij}}=\frac{a_{\mathrm{ij}}-\bar{a_{j}}}{B_{j}},\;i=1,2,3\ldots \ldots ..n;j=1,2,\ldots \ldots .,p\;$$

where,

$$\bar{a_{j}}=\sum_{i=1}^{n}\frac{a_{\mathrm{ij}}}{n},{\;B}_{j}^{2}=\frac{\sum_{i=1}^{n}{\left(a_{\mathrm{ij}}-\bar{a_{j}}\right)}^{2}}{n-1}$$

The overall standardized matrix is given by Equation (5),

(5) $$G={\left(g_{\mathrm{ij}}\right)}_{n*p}=\left(G_{1},G_{2},\ldots \ldots .G_{p}\right)$$

2. Compute the correlation-coefficient matrix as shown in the Equation (6),

(6) $$K=\frac{1}{n-1}=G^{t}\;G$$

where K represents (n∗n) symmetric matrix, data diagonal=1, $G^{t}$ indicates transposed-matrix G.

3. Compute eigenvalues and vectors of the correlation-coefficient matrix. Eigenvalues ($\gamma_{i}$) are procured by |γE−K|=0, sort $\gamma_{i}$ with size ($\gamma_{1}\geq \gamma_{2}\ldots \ldots \ldots \geq \gamma_{n}\geq 0$). Eigen vectors (${\mathrm{GA}}_{i}$) are attained by (γE−K)X=0, and$\;{\mathrm{GA}}_{i}={\left({\mathrm{GA}}_{i1},{\;\mathrm{GA}}_{i2},{\;\mathrm{GA}}_{\mathrm{ip}}\right)}^{t}$.

4. Determine the overall contribution of principal components as given by Equation (7),

(7) $$\beta_{i}=\frac{\gamma_{i}}{\sum_{i=1\;}^{p}\gamma_{i}}$$

The cumulative contribution of principal components (m) is given by Equation (8),

(8) $${\mathrm{CC}}_{i}=\frac{\sum_{i=1}^{m}\gamma_{i}}{\sum_{i=1}^{p}\gamma_{i}}$$

Usually, cumulative contribution ${\mathrm{CC}}_{i}\;\geq 85\%$ of principal components (m) are chosen.

5. Find the principal component as given by Equation (9),

(9) $$F_{i}={\mathrm{GA}}_{i1}\times G_{1}+{\mathrm{GA}}_{i2}\times G_{2}+\cdots +{\mathrm{GA}}_{\mathrm{ip}}\times G_{p}\;$$

6. Find the comprehensive-evaluation function given by Equation (10),

(10) $$F=\frac{\gamma_{1}F_{1}+\gamma_{2}F_{2}+\cdots +\gamma_{m}F_{m}}{\gamma_{1}+\gamma_{2}+\cdots +\gamma_{m}}$$

### 3.3. Classification Using M-RF (Modified-Random Forest) and NB, DT, and K-NN

Random Forests are generally an ensemble learning approach. The fundamental idea behind this algorithm is that the construction of small DT with minimum features seems computationally cheap. Moreover, when constructing several small and weak DTs in parallel, these trees can be integrated to attain strong learners by considering a majority vote. This work enhances the use of RFs for SSL (Semi-Supervised Learning) issues. From this insight, a multi-class marginal definition is developed for unlabeled data with an iterative deterministic annealing style training approach enhancing multiple class margins of unlabeled and labeled samples. Particularly, this permits using predicted labels corresponding to unlabeled data with additional variables for optimization. The main intention of this algorithm lies in learning weights on the data subset, which alleviates loss or error amongst predicted value and ground truth for precise classification of input to related label. In addition, the empirical loss function considered with RF computes the variation between predicted class and ground truth labels, termed cross-entropy. The loss value seems less when the prediction is near the ground-truth label. Contrarily, the resultant log loss seems to be high when the prediction seems to be beyond the true label. The overall process is shown in Algorithm 1.
**Algorithm 1:** Modified-Random ForestInput: Dataframe DFOutput:Class name(classification)BeginStep-1: data spitting=80:20training set=80;testing set=20Feat−F; number of trees in the forest−TStep-2: func RandomForestClassifier(DF, Feat)I−→∅for j∈1,…, B do${\mathrm{DF}}^{i}-a\;\mathrm{sample}\;\mathrm{from}\;\mathrm{DF}$$i_{j}-\mathrm{RandomizedTreeLearn}\left({\mathrm{DF}}^{i},\;\mathrm{Feat}\right)$$I-I\cup \left(i_{j}\right)$end forreturn Iend funcStep-3: func RandomizedTreeLearn(DF, Feat)at each node (R):Step-4: A loss function $\mathrm{elf}\left(g_{y}\left(x\right)\right)$ is claimed to be margin maximizing loss when $\mathrm{elf}\prime \left(g_{y}\left(x\right)\right)\leq 0$ for all${\;g}_{y}$ values. Hence, optimization relying on these loss function kinds will enhance the true margin. With maximization of the loss function ($\mathrm{elf}\left(g_{y}\left(x\right)\right)$, the local decision node ($R_{j}$) score is given by,${\mathrm{decision}\;\mathrm{node}\;R}_{j}=\mathrm{elf}\left(R_{j}\right)=\overset{K}{\underset{i=1}{\sum }}f_{i}^{j}l\left(f_{i}^{j}-\frac{1}{T}\right)$Step-5: s(f)=small subset of FeatStep-6: Split on the best Feat in the **s**(**f**)Step-7: return the learned treeend func

#### 3.3.1. NB (Naïve Bayes)

Naïve Bayes is a simple probabilistic and easy classifier that employs the Bayes theorem. This algorithm regards individual attribute variables as independent variables. Such a classifier could be trained efficiently in supervised learning. It could also be used in complex real-time circumstances. The main merit of NB is that it needs less training data for characterization. Due to these inherent advantages of NB, this study considers NB. Typically, classification is accomplished through the Bayes principle, and its overall process is shown in Algorithm 2.
**Algorithm 2:** Naïve BayesInput: Dataframe DFOutput:Class name(classification)Begin**Step-1:**data spitting=80:20training set=80;testing set=20**Step-2:**training settotal (t)=all samples in the training set DFprob=probability ;Frequency=f;$C_{j}\;\mathrm{is}\;\mathrm{the}\;\mathrm{class}\;\mathrm{in}\;\mathrm{the}\;\mathrm{training}\;\mathrm{set}$Calculate the prob of each class for classification$\mathrm{Prob}\left(C_{j}\right)=\frac{f\left(C_{j}\right)}{t}$Calculate the mean and standard deviationmean(m)= μ;standard deviation(sd)= σ;calculate m, sd for each feature in each class in DF and save it.**Step-3:**Testing setA sample in the testing set DFProbability density function (pdf)$\mathrm{pdf}\left(A\right){\;\mathrm{at}\;C}_{j}\;\mathrm{for}\;\mathrm{values}\;\mathrm{of}\;\mathrm{features}\;\mathrm{of}\;A.\;\mathrm{it}\;\mathrm{exists}\;\mathrm{in}\;\mathrm{the}\;\mathrm{DF}$$p(A_{i}|C_{j})$Calculating conditional probability ((cp)(A))${\mathrm{at}\;C}_{j}$result get by the below equation$P\left({A|C}_{j}\right)=\overset{n}{\underset{j=1}{\prod }}P(b_{j}|C_{j})$Calculating posterior probability((pp)(A)),$p\left(C_{j}|A\right)$$\left(\left(\mathrm{pp}\right)\left(A\right)\right),p\left(C_{j}|A\right)-\to {\mathrm{probability}\;\mathrm{of}\;\mathrm{samples}\;\mathrm{at}\;C}_{j}$$\mathrm{Assign}\;\mathrm{class}\;\mathrm{labels}\;\mathrm{to}\;\mathrm{the}\;\mathrm{class}\;\mathrm{of}\;A\;\mathrm{by}\;\mathrm{maxi}\left(p\right)=\left(C_{j}|A\right)$**Step-4:**Return classend

#### 3.3.2. DT (Decision Tree)

A Decision Tree remains a robust and renowned tool for prediction and classification. It is a flow chart-like structure wherein an individual internal node indicates the test on an attribute, an individual branch denotes the test outcome and an individual leaf node represents the class label. Moreover, DT classifies the instances by sorting from root to certain leaf nodes that afford instance classification. Constructing DT does not need any parameter setting or domain-oriented knowledge. Hence, it is suitable for exploratory knowledge discovery.

Furthermore, DTs could also handle data with high dimensions. The DT induction is a general inductive strategy for knowledge learning on classification. Due to such merits, this study considers DT for classification. Its overall process is shown in Algorithm 3.
**Algorithm 3:** Decision TreeInput: Dataframe DFOutput:Class name(classification)Begin**Step-1:**data spitting=80:20training set=80;testing set=20**Step-2:**finding→most repeated answer in the DF iflabel in the DF is unambiguous thenreturn Leaf(finding) \\return in case no further splitelse ifremaining features are empty thenreturn leaf (finding) \\quering more featureselsefor all Feat∈remaining features dono→subset of the DF on which Feat=noyes→subset of the DF on which Feat=yesvalue[Feat]→if highest vote in→no→if the highest vote is in→yesend for**Step-3:**Feat→the features with maximum values (Feat)no→subset of the DF on which Feat=noyes→subset of the DF on which Feat=yesrf=remaining featuresleft branch tree→decision tree (NO, rf)right branch tree→decision tree (Yes, rf)$\mathrm{return}\;\mathrm{Node}\left(\begin{array}{c} \mathrm{rf},\;\mathrm{left}\;\mathrm{branch}\;\mathrm{tree}\;\mathrm{right}\;\mathrm{branch}\\ \;\mathrm{tree} \end{array}\right)$end **Step-4:**return class (positive, negative, neutral)end

#### 3.3.3. K-NN (K-Nearest Neighbor)

K-Nearest Neighbor is a supervised simple ML algorithm that could be utilized for solving classification problems. It is easy to execute and comprehend. It attempts to determine the correct test data class by computing the distance between training and testing data. Subsequently, K-points are selected that are near to test data. K-NN computes the test data probability of K-training data classes and holds the maximum probability for selection. Due to these advantages, this study considers K-NN. The overall process of this algorithm is shown in Algorithm 4.
**Algorithm 4:** K-Nearest NeighborInput: Dataframe DFOutput:Class name(classification)Begin**Step-1:**data spitting=80:20training set=80;testing set=20**Step-2:**for each process X in the testing set, do Let X=X(1)+X(2) if X(1)≥X(2):X(1)(negative or neutral)if X(2)<X(1)X(2)(Normal)else then**Step-3:**${\mathrm{for}\;\mathrm{each}\;\mathrm{process}\;\mathrm{DF}}_{j}\;\mathrm{in}\;\mathrm{the}\;\mathrm{training}\;\mathrm{set},\;\mathrm{do}$${\mathrm{cal}\;\mathrm{depression}}_{\;\mathrm{features}}\left(\;X,{\;\mathrm{DF}}_{j}\right);$$\mathrm{if}\;\mathrm{dep}\left(X,{\mathrm{DF}}_{j}\right)\mathrm{equal}\;1.0\;\mathrm{then}$X(2) is normal ;exist;**Step-4:**Find k biggest scores of dep (X, DF);**Step-5:**${\mathrm{Calculate}\;\mathrm{dep}}_{\mathrm{avg}}\mathrm{fo}\;\mathrm{the}\;k-\mathrm{nn};$${\mathrm{if}\;\mathrm{dep}}_{\mathrm{avg}}>\mathrm{threshold}\;\mathrm{then}\;$X(1)−negative, neutralelse thenX(2)−normal

## 4. Results and Discussion

Outcomes attained after the execution of the proposed system are discussed in this section with dataset description, performance metrics, outcomes for performance, and comparative analysis.

### 4.1. Dataset Description

This dataset comprises EEG brainwave data administered with the original statistical extraction approach. Data was gathered from two individuals (one male and one female) for three minutes/state: positive, negative, and neutral. Muse EEG-headband that recorded AF8, TP10-EEG, AF7, and TP9 placements through dry-electrodes is utilized. Moreover, resting ‘neutral’ data is recorded for 6 minutes. The dataset is taken from, https://www.kaggle.com/datasets/birdy654/eeg-brainwave-dataset-feeling-emotions (accessed on 1 September 2022).

### 4.2. Performance Metrics

This section presents the main metrics regarded for this research and their corresponding mathematical representation.

(i) Accuracy—Ac

It is the computation of total-correct classification. It is given by Equation (11),

(11) $${\mathrm{Accuracy}}_{\mathrm{Ac}}=\frac{{\mathrm{Tr}}_{\mathrm{ntv}}+{\mathrm{Tr}}_{\mathrm{ptv}}}{{\mathrm{Tr}}_{\mathrm{ntv}}+{\mathrm{Fl}}_{\mathrm{ntv}}+{\mathrm{Tr}}_{\mathrm{ptv}}+{\mathrm{Fl}}_{\mathrm{ptv}}}$$

(ii) Precision—Pr

It is described as the overall correct classification, which is managed by misclassification. It is given by Equation (12),

(12) $${\mathrm{precision}}_{\mathrm{Pr}}=\frac{{\mathrm{Tr}}_{\mathrm{ptv}}}{{\mathrm{Tr}}_{\mathrm{ptv}}+{\;\mathrm{Fl}}_{\mathrm{ptv}}}$$

In Equations (11) and (12), ${\mathrm{Tr}}_{\mathrm{ntv}\;}$—true negative; ${\mathrm{Tr}}_{\mathrm{ptv}}—$true positive; ${\mathrm{Fl}}_{\mathrm{ntv}}$—false negative; and ${\mathrm{Fl}}_{\mathrm{ptv}}$—false positive.

(iii) Recall—Rc

It is stated as the proportion of relevant text and retrieved text to the proportion of the relevant text. It is given by Equation (13),

(13) $${\mathrm{Recall}}_{\mathrm{Rc}}=\frac{{\mathrm{Rlvt}}_{\mathrm{txt}}\cap {\mathrm{Retvd}}_{\mathrm{txt}}}{{\mathrm{Rlvt}}_{\mathrm{txt}}}$$

In Equation (13), ${\mathrm{Rvlt}}_{\mathrm{txt}}$—relevant text and ${\mathrm{Retv}}_{\mathrm{txt}}$—retrieved text

(iv) F-Measure—$F_{M}$

The F1-score is determined as the harmonic mean of recall and precision. It is given by Equation (14),

(14) $$F-{\mathrm{Measure}}_{F_{M}}=\frac{2*\left(\mathrm{Rc}*\mathrm{Pr}\right)}{\mathrm{Rc}+\mathrm{Pr}}$$

In Equation (14), Rc—recall, and Pr—precision

### 4.3. Performance Analysis

The performance of the proposed system is an evaluation of metrics such as accuracy, F1-score, recall, and precision. The corresponding outcomes are shown in this section. Initially, the proposed system is assessed, and the attained outcomes are shown in Table 1.

**Figure 2:**
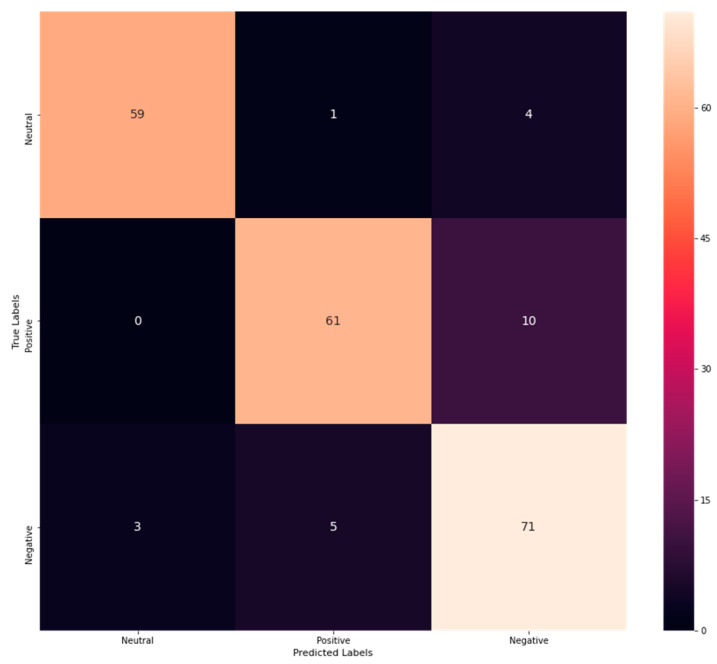
Confusion matrix of M-RF.

From Table 1, the accuracy rate of M-RF is exposed to be 0.98, precision as 0.98, recall as 0.96, and F1-score as 0.97. In addition, a confusion matrix is procured to explore correct and incorrect detection rates. The confusion matrix for the proposed M-RF is shown in Figure 2.

From Figure 2, it is clear that 59 emotions have been correctly classified as neutral, while three have been misinterpreted as negative. Similarly, 61 emotions have been correctly determined as positive, whereas one has been misinterpreted as neutral, and five have been misinterpreted as negative. On the contrary, 71 negative emotions have been correctly classified, 4 have been misinterpreted as neutral, and 10 have been misinterpreted as positive. Thus, from the analysis, it has been revealed that the correct classification rate is more than the misclassification, which exposes its better performance. Likewise, the performance of NB has been assessed through performance metrics. As a result, attained outcomes are explored in Table 2.

**Figure 3:**
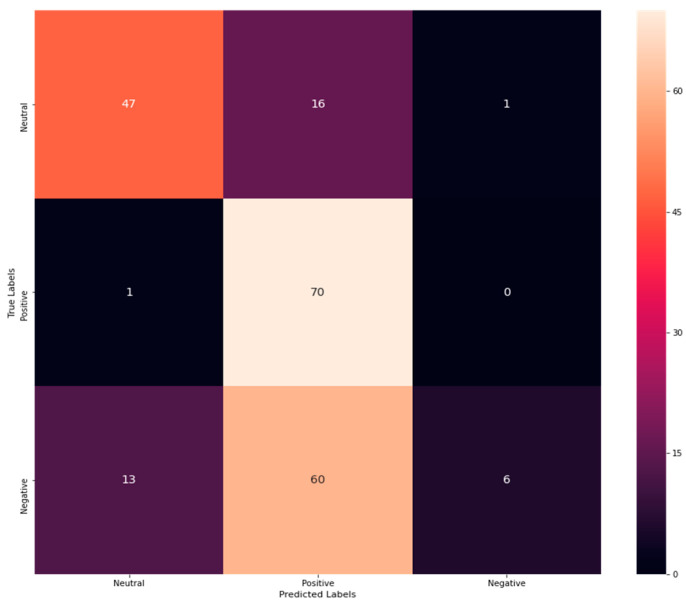
Confusion matrix of NB.

From Table 2, the accuracy rate of NB is exposed to be 0.574766355, precision as 0.7, recall as 0.6, and F1-score as 0.51. In addition, a confusion matrix is procured to explore correct and incorrect detection rates. The confusion matrix for NB is shown in Figure 3.

Figure 3 shows that 47 emotions have been correctly classified as neutral, while 13 have been misinterpreted as negative. Similarly, 70 emotions have been correctly determined as positive, 16 have been misinterpreted as neutral, and 60 have been misinterpreted as negative. On the contrary, six negative emotions have been correctly classified, while one has been misinterpreted as neutral. Thus, from the analysis, it has been revealed that the correct classification rate has been exposed to be more than the misclassification rate revealing its better performance. Similarly, the performance of DT has been evaluated by performance metrics. As a result, attained outcomes are explored in Table 3.

**Figure 4:**
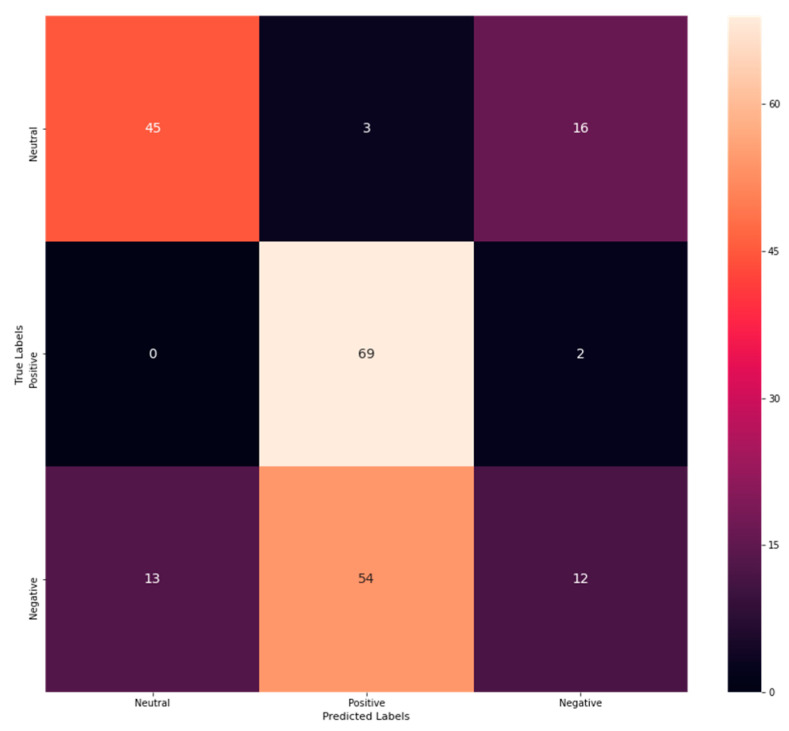
Confusion matrix of DT.

From Table 3, the accuracy rate of DT is exposed to be 0.588785046, precision as 0.57, recall as 0.61, and F1-score as 0.55. In addition, a confusion matrix is procured to explore correct and incorrect detection rates. The confusion matrix for DT is shown in Figure 4.

Figure 4 shows that 45 emotions have been correctly classified as neutral, while 13 have been misinterpreted as negative. Similarly, 69 emotions have been correctly determined as positive, 3 have been misinterpreted as neutral, and 54 have been misinterpreted as negative. On the contrary, 12 negative emotions have been correctly classified, while 16 have been misinterpreted as neutral and 12 as negative. Thus, from the analysis, it has been revealed that the correct classification rate has been exposed to be more than the misclassification rate revealing its better performance. Similarly, the performance of K-NN has been evaluated by performance metrics. As a result, attained outcomes are explored in Table 4.

**Figure 5:**
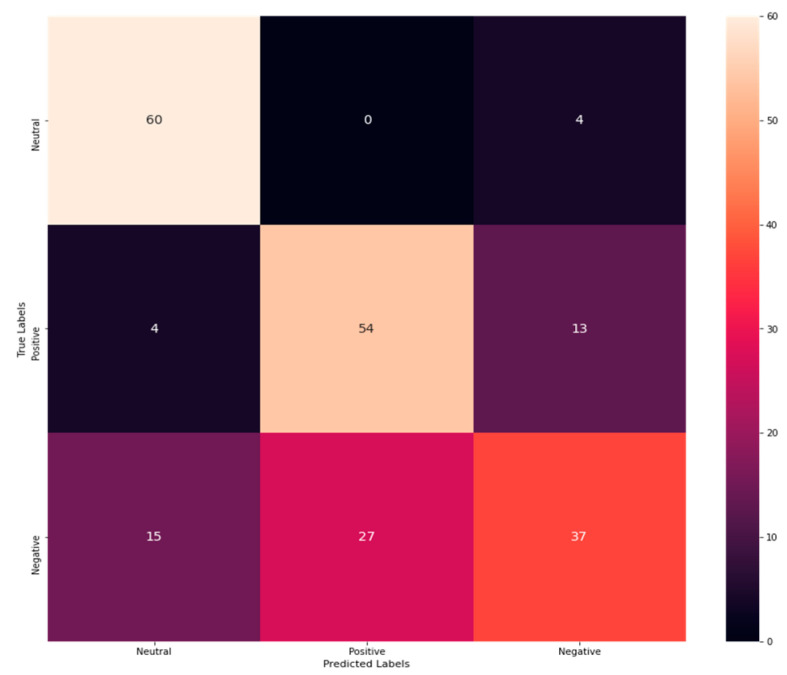
Confusion matrix of K-NN.

From Table 4, the accuracy rate of K-NN is exposed to be 0.705607476, Precision as 0.7, recall as 0.72, and F1-Score as 0.7. In addition, a confusion matrix is procured to explore correct and incorrect detection rates. The confusion matrix for K-NN is shown in Figure 5.

From Figure 5, it is clear that 60 emotions have been correctly classified as neutral, 15 have been misinterpreted as negative, and 4 have been misinterpreted as positive. Similarly, 54 emotions have been correctly determined as positive, whereas 27 have been misinterpreted as negative. On the contrary, 37 negative emotions have been correctly classified, while 4 have been misinterpreted as neutral and 13 as positive. Thus, from the analysis, it has been revealed that the correct classification rate has been exposed to be more than the misclassification rate revealing its better performance. From the performance analysis of M-RF, NB, DT, and K-NN, the performance of M-RF is better than the other three exposing its outstanding performance in emotion detection with a high rate of correct classification than misclassification rate.

### 4.4. Comparative Analysis

The proposed system has been comparatively evaluated with three conventional studies, and the corresponding outcomes are exposed in this section. Initially, analysis has been undertaken with conventional methods, namely DNN + Sparse Autoencoder (Deep Neural Network + Sparse Autoencoder), DCNN (Deep Convolutional Neural Network), Multi Column CNN, RNN (Recurrent Neural Network), LSTM (Long Short-Term Memory), and GRU (Gated Recurrent Unit). Obtained results are shown in Table 5.

From Table 5, it has been exposed that existing RNN has shown 0.97 as precision, and GRU has shown 0.97 as precision rate. At the same time, most methods have yet to be assessed precision and F1-score. However, the proposed system has evaluated the precision that has exposed 0.98 as precision. Likewise, existing RNN has shown 0.93 as F1-sScore, GRU has shown 0.95 as F1-score, and the proposed system has exposed 0.97 as F1-score. Moreover, the accuracy rates of existing algorithms such as DCNN have exposed 0.85, RNN has shown 0.92, GRU has exposed 0.95, and the proposed system has revealed 0.98. Thus, it is clear that the proposed system has exposed better performance than conventional algorithms. In addition, analysis has been undertaken with existing research encompassing LSTM and Devo MLP. As a result, attained results have been exposed in Table 6.

As shown in Table 6, analysis has been performed about accuracy for three emotion states, namely MindBigDataDigits, mental state, and emotional state. Outcomes have exposed that existing LSTM has shown better performance in classifying mental states with 84.44% accuracy. In contrast, LSTM has revealed a better performance of 97.06% for classifying emotional states, while existing Devo MLP attained 31.35% accuracy while classifying MindBigDataDigits. Though conventional algorithms have shown better performance, the proposed system has revealed superior performance with 98% accuracy in classifying the three emotion classes. Additionally, analysis has been undertaken with existing research. Attained results have been exposed in Table 7.

From Table 7, it has been explored that, among the considered existing algorithms SoftMax classifier has exposed better classification performance with 75.42% accuracy. However, the proposed system has explored superior performance with 98% accuracy. This better performance has been due to the proposed attention module that possessed the ability for consistent enhancement.

In addition, the proposed M-RF with empirical loss function could alleviate loss or error that have led the proposed system to explore effective classification, confirmed by the results. Further, an internal comparison has been undertaken by comparing the proposed M-RF classifier with ML classifiers namely NB, K-NN, and DT. The results are exposed in Table 8.

From Table 8, it has been revealed that, M-RF with empirical loss functions has shown 0.98 as accuracy; while, NB has exposed 0.57, DT has shown 0.58, whereas, K-NN has revealed 0.70. Furthermore, the precision rate of M-RF has been found to be 0.98, K-NN has shown 0.7, DT has revealed 0.57, while, NB has shown 0.7. Similarly, the recall and F1-score of the proposed M-RF is found to be better than other considered ML classifiers. The theoretical discussions to expose superior performance of the proposed system than the ML classifiers [36] are presented below.

Typically, NB algorithm has the ability to work fast and easily detect the test dataset class. It is also capable of solving multi-class prediction issues as it seems to be valuable. This algorithm exposes better classification performance when the assumption of independent features hold. In spite of its better performance, NB makes an assumption that all the features are unrelated or independent, so it is not capable of learning relationships amongst the features. 

In addition, K-NN might seem to be easy for execution, however, with increase in dataset, speed, and efficacy of the algorithm declines fast. Furthermore, K-NN functions better with less input variables; however, as the variables increase, K-NN encounters greater difficulty in predicting the outcomes of new data-point.

Furthermore, the DT algorithm averts the need for feature transformation when handling non-linear data as DTs do not consider multiple weighted integrations concurrently. DT is typically used for solving regression and classification issues. However, the main pitfall of this algorithm is that it typically results in data overfitting. 

Finally, in the present study, the proposed Deep Normalized Attention-based Residual CNN extracts significant and relevant features with minimum variance within it. These features are later passed into PCA to reduce dimensionality with less information loss. This process will also alleviate the complexity of the model and remove data noise. Dimensionality reduction assists in mitigating overfitting. As the RF typically seems to be biased while handling categorical variables, the proposed system employs an empirical loss function on RF (Random Forest) which makes it capable of accomplishing reliable and accurate outcomes when compared with conventional algorithms due to the optimized loss-variations undertaken with the proposed system. Based on these advantages, the performance gets enhanced in classifying emotions. The stepwise advantages of the proposed system are discussed below,

In this study, the proposed Deep Normalized Attention-based Residual CNN encompasses of three dense blocks, after which an attention block and an improved extraction layer with an activation function exist. Initially, present structure of dense connection is simplified. Later, an RCNN is proposed for the system to emphasize the local semantics which are related to emotion features. This enhanced framework is considered for refining the extracted convolutional-features and emphasizes that dense blocks possess fewer parameters while managing discriminative convolutional-feature indication ability. 

Furthermore, the proposed Modified Random Forest with Empirical loss function assigns attention weights along with parameters for DTs in a particular manner. Weights rely on distance amongst instance that falls into respective tree leaf. The proposed loss function specifically assists in computing weights by resolving the linear optimization issues. By these mechanisms, the proposed model gains the ability to learn and perform accurate prediction in comparison to traditional studies.

## 5. Conclusions

This study intended to classify emotions from the EEG dataset. To perform this, the study proposed DNA-RCNN for feature extraction, PCA for dimensionality reduction, and M-RF with empirical loss function for classification. Additionally, the study considered three ML classifiers—NB, DT, and K-NN—for internal comparison with M-RF. The confusion matrix was also explored to show the correct classification and misclassification rates. Performance was also assessed about performance metrics. From the analytical outcomes, M-RF exposed better performance than NB, DT, and K-NN, with a high rate of correct classification and a low misclassification rate. To further confirm the better performance of the proposed system, a comparative analysis was also undertaken with three traditional researchers. Outcomes confirmed that the proposed system explored superior performance in classifying emotions than conventional research with a 98% accuracy rate. In the future, advanced fusion methodologies could be utilized by varied feature kinds. For further enhancement, numerous features could be integrated into the system.

## Figures and Tables

**Table 1:** Analysis of proposed Modified-Random Forest.

	Accuracy	Precision	Recall	F1-Score
Modified-Random Forest with Empirical loss function	0.98	0.98	0.96	0.97

**Table 2:** Analysis of Naïve Bayes.

	Accuracy	Precision	Recall	F1-Score
Naïve Bayes	0.574766355	0.7	0.6	0.51

**Table 3:** Analysis of Decision Tree.

	Accuracy	Precision	Recall	F1-Score
Decision Tree Classifier	0.588785046	0.57	0.61	0.55

**Table 4:** Analysis of K-Nearest Neighbor.

	Accuracy	Precision	Recall	F1-Score
K-NN classifier	0.705607476	0.7	0.72	0.7

**Table 5:** Comparative analysis of performance metrics [33].

	Network	Precision	F1 Score	Accuracy
Existing network	DNN+ Sparse Autoencoder	NA	NA	0.96
	DCNN	NA	NA	0.85
	Multi column CNN	NA	NA	0.9
	3D CNN	NA	NA	0.88
	RNN	0.97	0.93	0.92
	LSTM	0.98	0.96	0.95
	GRU	0.97	0.95	0.95
	Proposed network	0.98	0.97	0.98
Proposed network	Proposed network	0.98	0.97	0.98

**Table 6:** Analysis of accuracy [34].

	Accuracy (%)		
	Mental State	Emotional State	Mind Big Data Digits
Devo MLP	79.7	96.23	31.35
LSTM	84.44	97.06	994
Proposed network	98		

**Table 7:** Analysis of accuracy [35].

Methods	Accuracy (%)
KNN	66.16
Decision Tree	60.36
Supervised Vector Machine	68.29
SoftMax classifier	75.42
Proposed method	98

**Table 8:** Analysis of M-RF classifier with other ML classifiers (NB, K-NN, and DT).

Algorithm	Accuracy	Precision	Recall	F1-Score
Modified-Random Forest with Empirical Loss Function	0.98	0.98	0.96	0.97
Naïve Bayes	0.5747664	0.7	0.6	0.51
Decision Tree Classifier	0.588785	0.57	0.61	0.55
K-NN Classifier	0.7056075	0.7	0.72	0.7

## Data Availability

The data that support the findings of this study are available on request from the corresponding author.
